# Relapsing Polychondritis following Treatment with Secukinumab for Ankylosing Spondylitis: Case Report and Review of the Literature

**DOI:** 10.1155/2018/6760806

**Published:** 2018-07-02

**Authors:** Alexander Zheutlin, Elena Schiopu

**Affiliations:** University of Michigan Medical School, Ann Arbor, MI, USA

## Abstract

Relapsing polychondritis (RP) is an autoimmune disorder that often occurs concomitantly with other autoimmune diseases, though RP has been infrequently associated with ankylosing spondylitis (AS). There is a small, but growing, body of the literature demonstrating case reports describing RP secondary to AS in patients treated with tumor necrosis alpha inhibitors (TNFi's). We present the first case in which RP developed in AS while treated with an interleukin 17A inhibitor (IL-17Ai), secukinumab. With this case report, we hope to raise physician awareness of the possible autoimmune disorders that may arise subsequent to novel immunomodulation therapies, particularly that RP may develop subsequent to inhibition of IL-17A.

## 1. Introduction

Relapsing polychondritis (RP) is classified as a rare disease by the National Organization for Rare Disorders, with an incidence of 3.5 per 1,000,000 and has a paucity of research elucidating a clear etiology [1]. RP is an autoimmune disease, which targets the cartilaginous framework in multiple organ systems, but demonstrates a predilection for the ears, nose, and larynx [2]. RP is documented in patients with other autoimmune diseases as frequently as 30–37% of the time [3]. The association between RP and AS has been reported in only few cases over the many decades of study. However, recent work estimates that as many of 12% of patients with AS have concomitant RP, with a risk of lifetime comorbidity risk of 67% to develop RP [4]. Historical treatment of AS focused on nonsteroidal anti-inflammatory drugs (NSAIDs), with more recent therapeutic regimens including TNFi. Recently, a human monoclonal antibody targeting IL-17A, secukinumab, has demonstrated efficacy in AS patients who failed a trial of TNF-alpha inhibitors, and for those who have never utilized any other biologic drugs [5]. Of the major clinical trials examining secukinumab, side effects were found to be similar to the placebo group, with nasopharyngitis and inflammatory bowel disease (IBD) being the most common adverse event [5]. Given the recent availability of secukinumab, no research has yet associated with antibody formation and newly developed autoimmune disease with the IL-17A inhibitors. The current case is the first report of RP development secondary to the use of an IL-17Ai for treatment of AS.

## 2. Case Report

Our patient, M.J., is a 56-year-old male, who has had inflammatory back pain since his twenties, but was diagnosed with AS at 53 years while hospitalized for small bowel obstruction. He was found to have sacroiliitis, enthesitis, inflammatory arthritis, positive HLA-B27, and elevated C-reactive protein (CRP) at 2.1 mg/dl (normal < 0.6 mg/dl).

**Figure 1:**
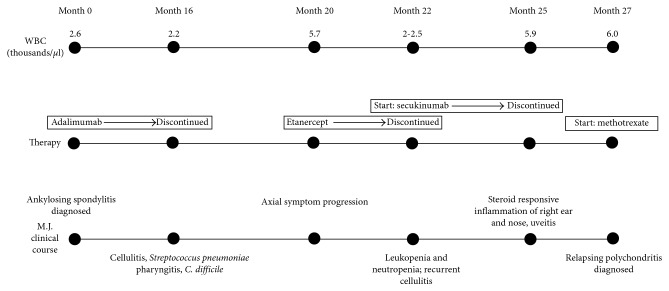
Clinical course of patient, M.J. The three timelines overlap laboratory findings with therapeutic regimen and clinical course.

At the time of diagnosis, M.J. was started on adalimumab 40 mg subcutaneously once every 14 days and celecoxib as needed. Despite an initial positive symptomatic response, his axial manifestations persisted, and he developed peripheral inflammatory arthritis in ankles, feet, wrists, and metacarpophalangeal (MCP) joints. At 18 months after initiation of adalimumab, the patient developed leukopenia and neutropenia, associated with mild infections such as cellulitis and gastroenteritis. Adalimumab was held for 6 months, and etanercept was initiated due to AS flares. After 3 months of symptomatic relief and adequate disease control, he developed leukopenia and etanercept was subsequently discontinued. At the time the leukopenia occurred, the patient did not have clinical manifestations of drug-induced SLE (rash, arthritis, hypocomplementemia, or proteinuria/hematuria); he was found to have +ANA (1 : 160, homogeneous pattern) and negative double-stranded DNA. A thorough hematological workup ruled out any other causes of leukopenia, and a decision was made to avoid TNFi's and to start the patient on secukinumab (complete clinical course is shown in Figure 1).

Secukinumab was started with an initial loading dose of 150 mg subcutaneously weekly for five weeks, followed by monthly doses. Following the last loading dose, the patient had an episode of gastroenteritis which was treated with 7 days of ciprofloxacin, and developed swelling, erythema, and throbbing pain of his bilateral ears and tip of the nose. He started a 17-day course of intravenous daptomycin and ertapenem, as his symptoms were thought to be secondary to a neutropenic infection. However, his symptoms did not abate. The patient developed periorbital edema and uveitis, which resolved with topical steroids. Additionally, he was started on 60 mg of daily prednisone by his primary care provider, tapered to 20 mg daily within a week, with resolution of his swelling and pain.

**Figure 2:**
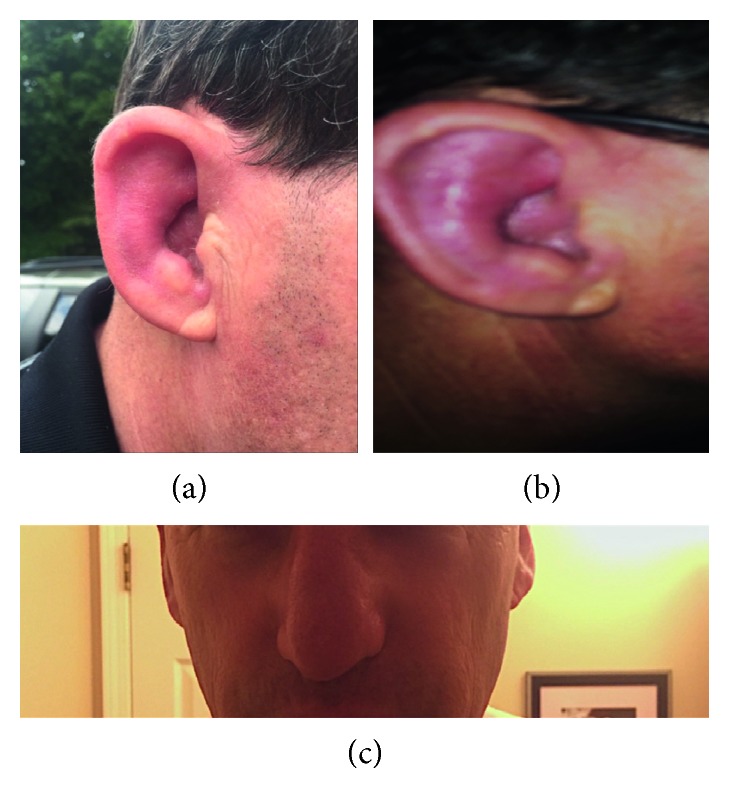
Panel image demonstrating new-onset auricular chondritis and hyperemia in the above two panels, with nasal chondritis visualized in the bottom panel.

Upon physical examination in our rheumatology clinic (four days after the steroids were stopped), there was nasal erythema diffusely, with mild tenderness to palpation. Bilateral auricular chondritis was present, with moderate hyperemia of the right ear (Figure 2). Some cartilaginous collapse was noted as well. Additionally, the patient had mild anterior uveitis present on the lateral aspect. At this time, the patient had a positive Schober's test of 13.5 centimeters, and no synovitis of the appendicular joints was noted. Based upon the presence of bilateral auricular chondritis, nasal chondritis, and recent ocular inflammation, RP was diagnosed based upon clinical presentation and history.

The patient was started on oral prednisone 20 mg for seven days, with a reduction of 5 mg per week as tolerated, along with 20 mg of methotrexate once a week, with a folic acid supplement of 1 mg. Following initiation of prednisone, there was resolution of the clinical manifestation of RP (auricular and nasal hyperemia and chondritis, as well as uveitis) along with improvement in the inflammatory markers.

## 3. Discussion

The Assessment of Spondyloarthritis treatment guidelines include a trial of NSAIDs for four weeks, following by TNFi's in the event NSAIDs do not provide clinical improvement [6]. However, numerous patients do not achieve remission with initial TNFi therapy [7]. This poses a problem in treatment, as a trial of a different TNFi is suggested following the failure of a first TNFi. Recent clinical trials examining new IL-17Ai have demonstrated their utility in achieving clinically significant improvement in patients with AS and were approved by the FDA in January of 2016 [8, 9]. IL-17Ai has good tolerability, with a side effect distribution similar to the effect of placebo. Notable side effects found in the MEASURE 1 and MEASURE 2 trials were nasopharyngitis, headache, viral infection, dyslipidemia, nausea, influenza, and mouth ulcers [5].

RP is considered an autoimmune disease with a reaction to endogenous type II collagen [10]. Chondrocytes are targeted by antibodies becoming necrotic before being replaced with fibrotic cell lineages [11]. The current paradigm for the pathogenesis of RP involves cytokine-mediated immunologic activity via IL-17A and TNF-alpha leading to matrix-degrading proteinases production from chondrocytes [12]. Additionally, antibodies to type II collagen and CD4+ cells have been implicated in the disease pathogenesis, though the exact relationship is not clearly known [13]. These underlying biochemical processes lead to the clinical manifestations of auricular chondritis, nasal chondritis, laryngeal chondritis, nondeforming or erosive arthritis, and various ocular manifestations, including uveitis.

Various treatment modalities have been found to be effective in treating RP. Patients with mild inflammation can be treated with NSAIDs and low-dose prednisone. Dapsone or higher prednisone doses can be utilized in patients with more severe symptoms. In patients for whom an effective dose of steroids is not an option, methotrexate or azathioprine may be used to reduce the necessary burden of steroid therapy [2].

RP has been sparingly diagnosed in the context of TNFi therapy for patients with AS. In 2014, Azevedo et al. described a case of etanercept-induced RP in the treatment of an adult man with AS. The patient was HLA-B27 positive and was diagnosed based upon clinical suspicion two months after initiating etanercept therapy. This patient was taken off the TNF-alpha inhibitor and with the addition of corticosteroids saw improvement in his RP within five months [14]. Two similar cases were described by Hernández et al. in 2011, in which two HLA-B27 were diagnosed with newly developed RP as consequence of TNA-alpha inhibitor therapy [15]. These cases similarly presented with RP after approximately two months on TNFi. Steroids were started in each patient in combination with TNFi cessation, with resolution of symptoms after five to six months. TNFi's were successfully restarted without documented recurrence of RP [14, 15].

To our knowledge, this is the first described case of RP induced by IL-17Ai therapy. Our patient never presented with symptoms of RP prior to initiation of the IL-17Ai. Though a different agent, the clinical course is similar to cases in the literature documenting RP following TNF-alpha inhibitors. Autoantibody development seen in patients treated with TNF-alpha inhibitors may follow a similar pathogenesis as the clinical case documented in the current report. TNF-alpha and IL-17A are proinflammatory cytokines involved in the same pathway [16]. This pathway is a particularly exciting target for AS treatment. When compared to healthy controls, patients diagnosed with AS exhibit significantly higher levels of serum IL-17A [17]. Designing therapies to inhibit IL-17A may be of even more benefit in HLA-B27-positive patients. Misfolded HLA-B27 has been introduced as an important factor in upregulation of Th17 cytokines, including IL-17A [18].

The expression of IL-17A in a pathogenic context arises predominantly from a subset of Th cells, Th17. These cells also express TNF [19]. IL-17A and TNF have been suggested as synergistic inflammatory factors [20]. The dual effect of these cytokines can cause damage particularly to cartilage and bone [21]. These two cytokines augment inflammation via an increase in endothelial selectins for neutrophil chemotaxis as well as expression of neutrophil chemokines [22]. The balance of these cytokines is altered by biologic drugs. Patients who respond to TNFi decrease endogenous levels of IL-17A and TNF-alpha. However, in nonresponders to TNFi, there is a paradoxical elevation of Th17 and IL-17 [23].

The novel therapeutic targeting of IL-17A is attractive due to the relationship between AS and poorly modulated IL-17A production, and the ability of IL-17 inhibitors to diminish overexpression of IL-17 [8]. Additionally, the effectiveness as studied in the MEASURE 1 and 2 trials demonstrates the powerful utility of this class of drugs in achieving clinical remission [5]. The development of RP and paradoxical increase in inflammation of cartilage secondary to IL-17A inhibitors is likely the result of a disturbance in the equilibrium of these cytokines. Blocking IL-17A cytokines allows for the potential of increasing other inflammatory cytokines, such as IL-17F, which can act at the same receptor, and TNF-alpha [24]. There is still an incomprehensive understanding of the causal relationship between TNFi's and IL-17A inhibitors with RP development, necessitating further study to elucidate the relationship.

## 4. Conclusion

This is the first reported case of RP following treatment with an IL-17A inhibitor, adding to a growing body of evidence emphasizing new onset of autoimmune diseases in a subset of patients. Prior cases have been found subsequent to TNFi use and have increased clinician awareness of the potential development of RP in patients with AS. The clinical diagnosis of RP in the current case is supported by clinical evidence of polychondritis and elevated inflammatory markers, as well as symptom resolution following the discontinuation of secukinumab and initiation of prednisone therapy. Given the novelty of IL-17Ai and restricted treatment options of AS, it is important that physicians are wary of the potential development of RP following IL-17Ai use in patients with AS.

## References

[1] O’Fallon W. M., McKenna C. H., Michet J. (1986). Relapsing polychondritis. Survival and predictive role of early disease manifestations. *Annals of Internal Medicine*.

[2] Le C., Trentham D. (1998). Relapsing polychondritis. *Annals of Internal Medicine*.

[3] Letko E., Zafirakis P., Baltatzis S. (2002). Relapsing polychondritis: a clinical review. *Seminars in Arthritis and Rheumatism*.

[4] Horvath A., Pall N., Molnar K. (2016). A nationwide study of the epidemiology of relapsing polychondritis. *Clinical Epidemiology*.

[5] Blair H. A., Dhillon S. (2016). Secukinumab: a review in ankylosing spondylitis. *Drugs*.

[6] Braun J., Dougados M., Burgos-Vargas R. (2011). 2010 update of the International ASAS recommendations for the use of anti-TNF agents in patients with axial spondyloarthritis. *Annals of the Rheumatic Diseases*.

[7] Arends S., Spoorenberg A., Brouwer E. (2012). Baseline predictors of response to TNF-α blocking therapy in ankylosing spondylitis. *Current Opinion in Rheumatology*.

[8] Baeten D., Baraliakos X., Braun J. (2013). Anti-interleukin-17A monoclonal antibody secukinumab in treatment of ankylosing spondylitis: a randomised, double-blind, placebo-controlled trial. *The Lancet*.

[9] Baeten D., Sieper J., Braun J. (2015). Secukinumab, interleukin-17A inhibition in ankylosing spondylitis. *New England Journal of Medicine*.

[10] Deodhar S. D., Bergfeld W. F., Valenzuela R. (1980). Relapsing polychondritis. Immunomicroscopic findings in cartilage of ear biopsy specimens. *Human Pathology*.

[11] Costedoat-Chalumeau N., Guillevin L., Terrier B. (2014). Relapsing polychondritis. *Joint Bone Spine*.

[12] Shimizu J., Oka H., Yudoh K. (2016). Cutaneous manifestations of patients with relapsing polychondritis: an association with extracutaneous complications. *Clinical Rheumatology*.

[13] Kemta L. F., Kraus V. B., Chevalier X. (2012). Biologics in relapsing polychondritis: a literature review. *Seminars in Arthritis and Rheumatism*.

[14] Azevedo V. F., Galli N. B., Kleinfelder A. D. F. (2014). Relapsing polychondritis in a patient with ankylosing spondylitis using etanercept. *Case Reports in Rheumatology*.

[15] Hernández M. V., Ruiz-Esquide V., Gómez-Caballero M. E. (2011). Relapsing polychondritis: a new adverse event secondary to the use of tumour necrosis factor antagonists?. *Rheumatology*.

[16] Coats I., Suarez-Farinas M., Pierson K. C. (2008). Th17 cytokines interleukin (IL)-17 and IL-22 modulate distinct inflammatory and keratinocyte-response pathways. *British Journal of Dermatology*.

[17] Wendling D., Racadot E. (2006). Serum tissue factor levels correlate with inflammation in ankylosing spondylitis. *Joint Bone Spine*.

[18] Turner M. J., Sowders D. P., DeLay M. L. (2005). HLA-B27 misfolding in transgenic rats is associated with activation of the unfolded protein response. *Journal of Immunology*.

[19] Wang Y., Park H., Yang X. O. (2005). A distinct lineage of CD4 T cells regulates tissue inflammation by producing interleukin 17. *Nature Immunology*.

[20] Kirkwood K. L., Wong G. C., Kasayama S. (2004). Functional cooperation between interleukin-17 and tumor necrosis factor-alpha is mediated by CCAAT/enhancer-binding protein family members. *Journal of Biological Chemistry*.

[21] Jones S. A., Sutton C. E., Cua D. (2012). Therapeutic potential of targeting IL-17. *Nature Immunology*.

[22] Griffin G. K., Newton G., Tarrio M. L. (2012). IL-17 and TNF-α sustain neutrophil recruitment during inflammation through synergistic effects on endothelial activation. *Journal of Immunology*.

[23] Xueyi L., Lina C., Zhenbiao W. (2013). Levels of circulating Th17 cells and regulatory T cells in ankylosing spondylitis patients with an inadequate response to anti-TNF-[alpha] therapy. *Journal of Clinical Immunology*.

[24] Zhu S., Qian Y. (2012). IL-17/IL-17 receptor system in autoimmune disease: mechanisms and therapeutic potential. *Clinical Science*.

